# Maintained growth performance and reduced mortality of genetically resistant nursery pigs after an experimental virulent F18 enterotoxigenic *Escherichia coli* challenge

**DOI:** 10.1093/tas/txaf004

**Published:** 2025-01-17

**Authors:** Michael W Welch, Amanda J Cross, Iara D P Solar Diaz, Danielle C Johnson, Eric Parr, Tom A Rathje, Randy C Borg, Dustin D Boler

**Affiliations:** Carthage Veterinary Service Ltd, Carthage, IL 62321, USA; DNA Genetics, Columbus, NE 68601, USA; DNA Genetics, Columbus, NE 68601, USA; Carthage Veterinary Service Ltd, Carthage, IL 62321, USA; Carthage Veterinary Service Ltd, Carthage, IL 62321, USA; DNA Genetics, Columbus, NE 68601, USA; DNA Genetics, Columbus, NE 68601, USA; Carthage Veterinary Service Ltd, Carthage, IL 62321, USA

**Keywords:** challenge, enterotoxigenic, *Escherichia coli*, F18, genetic resistance, pig

## Abstract

Enterotoxigenic *Escherichia coli* (ETEC) is a leading cause of postweaning diarrhea (PWD) and mortality of weaned pigs. The objective of this study was to evaluate genetic resistance of the polymorphism at nucleotide 307 (M307) in the *FUT1* gene, to F18 *E. coli* infection considering different genotypes. A total of 179 pigs were used for this study. Pigs were genotyped for susceptibility to F18+ *E. coli* prior to the trial. Treatments included: genotype M307^GA^—heterozygous for *E. coli* susceptibility (A), genotype M307^GG^—homozygous *E. coli* susceptibility (B), or genotype M307^AA^—homozygous for *E. coli* resistance (C). Pigs were weighed, assigned to pens based on genotype, and allowed to acclimate for 3 d prior to the challenge. On days 4, 5, and 6, pigs were inoculated intraorally at the oropharynx with an F18+ *E. coli* isolate at a geometric mean concentration of 9.8 × 10^9^. Growth rate (average daily gain [ADG]), feed intake (average daily feed intake), and gain-to-feed ratio (G:F) were calculated by pen. All pigs were humanely euthanized at the end of the trial. Two fixed sections of ileum and distal jejunum were collected from a subpopulation and tested by in situ hybridization (ISH) to evaluate F18+ *E. coli* adherence. Fresh ileum samples were used for enumeration of F18, total *E. coli*, and total bacteria by real-time polymerase chain reaction. Mortality rates during the trial were 26.7% for genotype A, 18.3% for genotype B, and 0.0% for genotype C (*P *< 0.01). Starting weights prior to inoculation were not different (*P* = 0.29) among genotypes. Overall, pigs from genotype C grew 223 g/d faster (*P* = 0.04) than genotype A. Pigs from genotype C tended to grow 185 g/d faster (*P* = 0.09) than genotype B. G:F for genotype C (0.74) was 23% greater (*P *< 0.01) than G:F for genotype A (0.60) and tended to be 12% greater (*P* = 0.07) than genotype B (0.66). There were no differences in ADG or G:F between genotypes A and B. F18-specifc Cq units were decreased by 7.74 and 6.47 in genotypes A and B compared with genotype C (*P* ≤ 0.03). Signal by ISH was increased by 14.0-fold in genotype A compared with genotype C (*P* = 0.02). Adherence was not different among genotypes (*P* = 0.40). Genotype A had greater mortality and poorer growth performance than genotype B or C. Genotype C had no mortalities during the trial, grew faster, was more feed efficient, and had less F18 *E. coli* in the ileal mucosa compared with genotype A. Resistant genotypes provide an opportunity to reduce PWD and mortality due to an F18+ *E. coli* infection.

## Teaser

This study evaluated genetic resistance in nursery pigs against F18 *Escherichia coli* -associated postweaning diarrhea after an experimental inoculation. Pigs with the homozygous resistant genotype had no mortalities during the trial, grew faster, were more feed efficient, and had fewer bacteria in the ileum compared to the homozygous susceptible genotype.

## INTRODUCTION

Enterotoxigenic *Escherichia coli* (ETEC) infection is a leading cause of diarrhea and nursery mortality. Lactogenic passive protection decreases at weaning due to decreasing maternal antibodies, and most postweaning diarrhea (PWD) associated with ETEC occurs within 2 wk postweaning (Deprez et al., 1986; Fairbrother et al., 2005). The majority of ETEC isolates associated with PWD almost exclusively express either the K88 (F4) or F18 fimbrial adhesin (Fairbrother et al., 2005). Since 2019, there has been a substantial increase in the frequency of detection of F18-positive isolates from cases with a positive ETEC diagnosis submitted to the Iowa State University Veterinary Diagnostic Laboratory compared to K88 (Paiva et al., 2024). In addition, antimicrobial resistance (AMR) to fluoroquinolones, an antibiotic class important in human medicine, has dramatically increased in F18-positive isolates beginning in 2017 (Paiva et al., 2024).

Mortality associated with PWD can increase from 2% to 7% following an ETEC outbreak, with mortality reaching 20% to 30% in some cases (Amezcua et al., 2002). Experimentally, inoculation with F18+ *E. coli* is reported to reduce daily gain by 50% and feed intake by as much as 30% (Becker et al., 2020). An increase in wean-to-finish mortality from 2% to 7% could cost a standard production system $3.85 per pig or $9,248 per 2,400 pig wean-to-finish farm, assuming a decrease in average dead weight from 68 to 27 kg, a wean pig purchase price of $35 per pig, and a market price of $1.38 per kg carcass weight (Euken and Schulz, 2021).

Many mitigation strategies have been evaluated to reduce F18+ *E. coli*-associated PWD with varying levels of success including antimicrobials (Fairbrother et al., 2005), vaccination (Van den Broeck et al., 1999; Ruan et al., 2011; Hur and Lee, 2013), supranutritional levels of zinc oxide (Jang et al., 2023; Hansen et al., 2024), probiotics (Lessard et al., 2009; Duarte et al., 2020; Sun et al., 2021), and other dietary supplements (Becker et al., 2020; Smith et al., 2020; White et al., 2024). Host resistance to F18+ *E. coli* infection has gained increasing attention within the past 30 yr as another potential strategy to mitigate PWD. There have been several genetic loci thought to have a possible influence on F18+ *E. coli* adhesion and confer host resistance to F18+ *E. coli* infection (Meijerink et al., 1997; Frydendahl et al., 2003). The objective of this study was to quantify the level of disease susceptibility associated with three different host genotypes on PWD after experimental inoculation with a virulent F18+ *E. coli* isolate. The hypothesis was that pigs with a homozygous resistant genotype would have decreased mortality, improved clinical signs, and increased growth performance based on previous research (Frydendahl et al., 2003).

## MATERIALS AND METHODS

All materials and methods were approved by the Carthage Institutional Animal Care and Use Committee prior to starting the study (protocol 2023-31). Pigs were humanely euthanized when they showed at least one of the following clinical signs including but not limited to lateral recumbency or immobility, lack of response to stimulation, inability to eat or drink, or hypothermia.

### Experimental Design

A total of 179 market pigs (DNA Line 600 × DNA Line 241) were sourced from a high-health commercial research farm and transported to Carthage Innovative Swine Solutions Veterinary Research Facility (CISS-VRF) located near Champaign, IL. The commercial research farm was negative for common porcine viral pathogens including porcine epidemic diarrhea virus, porcine deltacoronavirus, transmissible gastroenteritis virus, and porcine reproductive and respiratory syndrome virus by routine testing. The sows were screened by real-time polymerase chain reaction (rPCR) and confirmed negative for the presence of F18+ *E. coli* from the candidate source litters 7 d prior to placement at the CISS-VRF as previously described (White et al., 2024). All pigs were genotyped with the high-density Affymetrix SusScrofa HD array that contains 66K SNPs prior to shipment. The polymorphism at nucleotide 307 (M307) was isolated and the animals were categorized as genotype M307^GA^—heterozygous for *E. coli* susceptibility (A), genotype M307^GG^—homozygous *E. coli* susceptibility (B), or genotype M307^AA^—homozygous for *E. coli* resistance (C). A total of 60 pigs, genotype A, 60 pigs, genotype B, and 59 pigs, genotype C were analyzed. Only 59 pigs were included in genotype C due to transport mortality. Pigs were weaned at 23 d of age as the F18 receptor has been shown to be adequately expressed in this age group (Coddens et al., 2007). All research staff at CISS-VRF who worked with the pigs or performed the analysis were blinded to pig genotype assignments. In order to account for family diversity, pigs were chosen randomly within each litter by genotype. Thus, 12 pigs were randomly allotted to pens by genotype so that only one genotype was represented per pen to allow for the calculation of mortality-adjusted growth performance characteristics including average daily gain (ADG), average daily feed intake (ADFI), and gain-to-feed ratio (G:F) on a pen basis. The pigs were provided an acclimation period of 3 d prior to the challenge to adjust to the facility. Individual pig weights were collected at −3, 3, 7, 17, and 35 days postinoculation (DPI). The pigs were all fed the same standard, commercial, mash diets in a three-phase nursery program upon arriving at CISS-VRF. Diets were manufactured at the University of Illinois Feed Technology Center. Phase changes occurred from phase 1 to phase 2 on 3 DPI and phase 2 to phase 3 on 17 DPI. The remaining feed was weighed, removed from the feeder, and recorded at each phase change and upon termination of the study (3, 17, and 35 DPI).

### Animals and Housing

The facility had four identical isolated production rooms containing five pens each measuring 3.35 m × 1.83 m with standard plastic flooring. Incoming air was filtered to comply with biosafety level 2 regulations and to maintain a pure challenge environment. Ventilation was regulated with an AeroSpeed 2.4 controller (AeroTech, Pittsburgh, PA). Setpoints were initially set at 28.5 °C at placement and decreased to 23.3 °C at the conclusion of the study. Nursery feeders were manufactured from stainless steel with two feeding spaces per pen. Ad libitum access to water was provided via a single-cup waterer per pen.

### F18+ *E. coli* Inoculation

All piglets were challenged with F18+ *E. coli* isolate 19-34773 obtained from the University of Illinois Veterinary Diagnostic Laboratory grown as previously described (White et al., 2024). Pigs were inoculated with a geometric mean concentration of 9.80 × 10^9^ colony forming units per milliliter (CFU/mL) or 9.8 × 10^9^ CFU/mL on 0 DPI, 7.63 × 10^9^ CFU/mL on 1 DPI, and 1.57 × 10^10^ CFU/mL on 2 DPI. The isolate expressed a heat-labile toxin, heat-stabile toxin B, Shiga-like toxin II, and heat-stabile enterotoxin. The inoculum was administered with a 3-mL syringe in the oral cavity at the level of the oropharynx to minimize spillage. All pigs were treated with an intramuscular injection of enrofloxacin (Baytril, Elanco, Greenfield, IN) at a dose of 7.5 mg/kg at 7 DPI due to high mortality and to ensure the welfare of the remaining pigs.

### Clinical Scoring

Clinical scores were collected daily from 0 to 14 DPI on an individual pig basis to evaluate the impact of F18+ *E. coli* inoculation on each genotype. Diarrhea score was measured by the following scale: 1—fully formed; 1—mild, soft; 2—mostly watery, some solids; 3—severe watery, no solids. Lethargy score was measured on the following scale: 1—normal, bright, alert, and responsive; 2—somewhat depressed, will stand; 3—severely lethargic/depressed. Emaciation score was measured on the following scale: 0—normal; 1—flank tucked in, good cover; 2—backbone showing; 3—ribs and backbone showing, emaciated.

### Sample Collection and Molecular Testing

Two pigs per pen were euthanized at 3 DPI (*n* = 10 pigs/group or *N* = 30). Two sections of ileum and two sections of jejunum were collected from each pig for evaluation by in situ hybridization (ISH) and one section of ileum was submitted for rPCR. Fixed tissues were placed in 10% neutral-buffered formalin for 24 h to ensure tissue fixation before being transferred to 70% isopropanol. An ISH assay was developed at the Iowa State University Veterinary Diagnostic Laboratory for visualization of adherent F18+ *E. coli* to the intestinal epithelium using a modified protocol as described previously (Arruda et al., 2019). DNA extraction and amplification for rPCR were performed at the University of Illinois Veterinary Diagnostic Laboratory as previously described (White et al., 2024).

### Statistical Analysis

Analyses were conducted in the open-source software R version 4.3.0 using the lm function and emmeans package (v1.8.5). Pig weights, ADG, ADFI, and G:F were analyzed on a pen basis by averaging the individual values by pen (when applicable) and adjusting for mortality events. ISH results were first log-transformed for normality. Both rPCR and ISH were analyzed on an individual pig basis. Clinical scores were averaged on a pen basis at each time point, and a mixed-model repeated measures approach was used for analysis with the mmrm package (v0.3.6). Independence, compound symmetry (CS), first-order autoregressive (AR1), heterogeneous first-order autoregressive (AR1H), and antedependence (ANTE) covariance structures were independently evaluated for each response. The covariance structure with the lowest Akaike’s information criterion was selected for each, including AR1H for both diarrhea and body condition scores and AR1 for lethargy score. Mortality by genotype was analyzed by a Kaplan–Meier plot using the survival package (v3.5-5) and Fisher’s exact test for cumulative mortality. The figures used in the manuscript were prepared using the ggplot2 package (v3.5.0). Statistical significance and marginal significance were considered at *P *<* *0.05 and 0.05 ≤ *P* < 0.10.

## RESULTS

### Mortality and Clinical Signs

**Figure 1.**
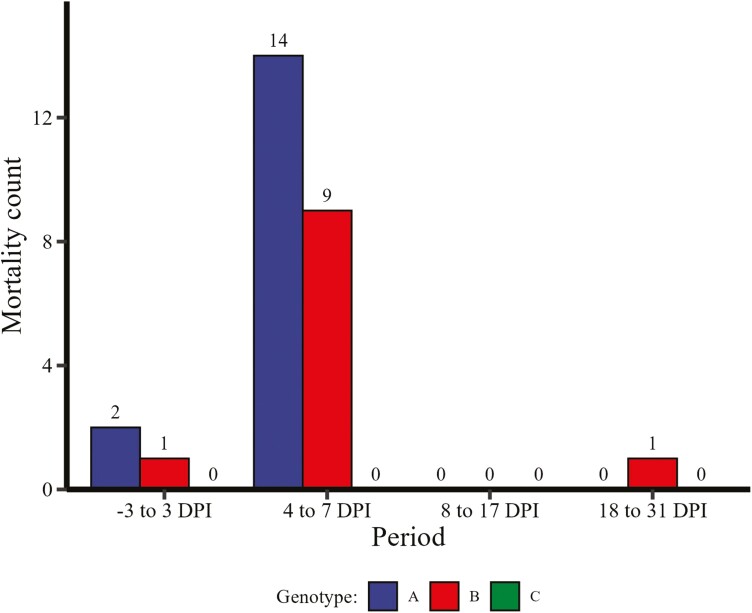
Number of pig deaths during each weighing period. The numbers above each bar are the death count during that period. Pig genotypes evaluated include A = genotype M307^GA^—heterozygous for *E. coli* susceptibility (*n* = 60, 5 pens, 12 pigs/pen), B = genotype M307^GG^—homozygous *E. coli* susceptibility (*n* = 60, 5 pens, 12 pigs/pen), and C = genotype M307^AA^—homozygous for *E. coli* resistance (*n* = 59, 5 pens, 11 to 12 pigs/pen). Pigs were orally inoculated with approximately 1 × 10^10^ CFU/mL for three consecutive days, starting at 0 DPI.

**Figure 2.**
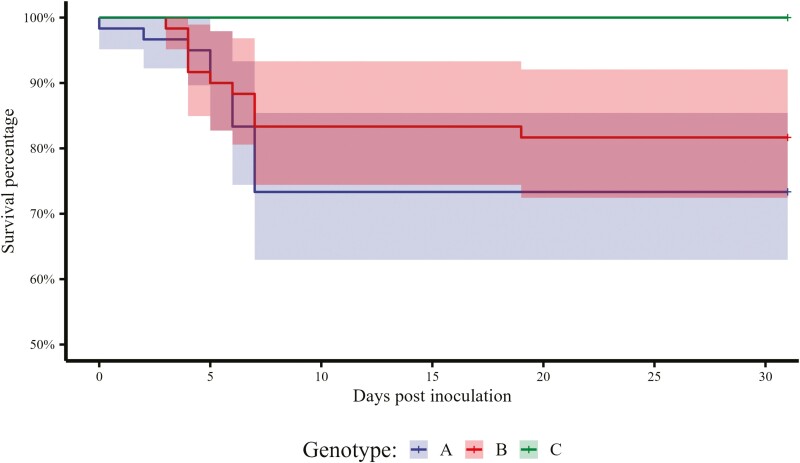
Kaplan–Meier plot of the percentage of weanling pigs surviving enterotoxigenic *Escherichia coli* oral inoculation. Pig genotypes evaluated include A = genotype M307^GA^—heterozygous for *E. coli* susceptibility (*n* = 60, 5 pens, 12 pigs/pen), B = genotype M307^GG^—homozygous *E. coli* susceptibility (*n* = 60, 5 pens, 12 pigs/pen), and C = genotype M307^AA^—homozygous for *E. coli* resistance (*n* = 59, 5 pens, 11 to 12 pigs/pen). Pigs were orally inoculated with approximately 1 × 10^10^ CFU/mL for three consecutive days, starting at 0 DPI.

There were no mortalities in any of the genotypes prior to inoculation (Figure 1). A total of two pigs died between 0 and 3 DPI from genotype A and one pig died from genotype B. No pigs died from genotype C. From 4 to 7 DPI, a total of 14 pigs died from genotype A, nine pigs died from genotype B, and no pigs died from genotype C. Only one pig died from genotype B in the subsequent weigh periods, and 0 pigs died from either genotypes A or C. Mortality was significantly reduced in genotype C (0.00%, 0/59) compared to both genotype B (18.33%, 11/60, *P* < 0.001) and genotype A (26.67%, 16/60, *P *< 0.001). There was no difference in mortality between genotype A genotype and genotype B (Figure 2, *P* = 0.38).

**Figure 3.**
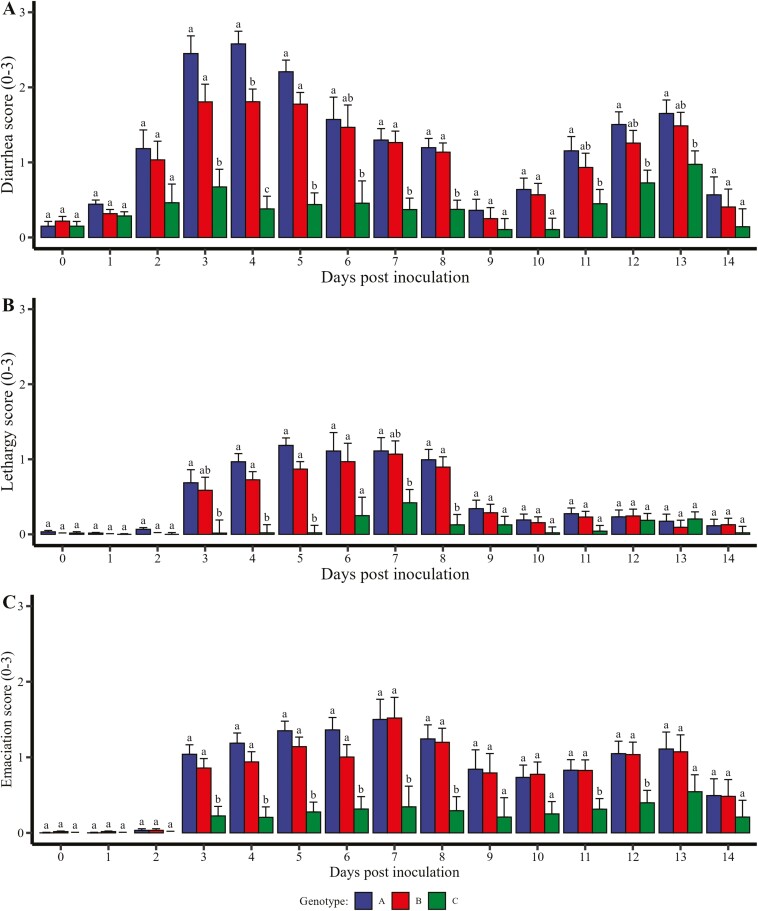
The severity of clinical signs was measured on an individual weanling pig basis from 0 to 14 DPI. The average disease score by pen was compared by genotype. Pig genotypes evaluated include A = genotype M307^GA^—heterozygous for *E. coli* susceptibility (*n* = 60, 5 pens, 12 pigs/pen), B = genotype M307^GG^—homozygous *E. coli* susceptibility (*n* = 60, 5 pens, 12 pigs/pen), and C = genotype M307^AA^—homozygous for *E. coli* resistance (*n* = 59, 5 pens, 11 to 12 pigs/pen). Pigs were orally inoculated with approximately 1 × 10^10^ CFU/mL for three consecutive days, starting at 0 DPI. (A) Diarrhea score was measured by the following scale: 1—fully formed; 1—mild, soft; 2—mostly watery; 3—severe watery, no liquids. (B) Lethargy score was measured on the following scale: 1—normal, bright, alert, and responsive; 2—somewhat depressed, will stand; 3—severely lethargic/depressed. (C) Emaciation score was measured on the following scale: 0—normal; 1—flank tucked in, good cover; 2—backbone showing; 3—ribs and backbone showing, emaciated.

Clinical scores followed the same directional trend as mortality (Figure 3). Genotypes A and B had more severe average diarrhea scores compared to genotype C (Figure 3A). Average diarrhea scores were significantly reduced by at least 0.68 units between genotype C relative to genotype A on 3, 4, 5, 6, 7, 8, 11, 12, and 13 DPI (*P* ≤ 0.05). There was a marginally significant reduction in average diarrhea scores of 0.53 units between genotype C relative to genotype A on 10 DPI (*P* = 0.07). In addition, average diarrhea scores were significantly reduced by at least 0.76 units between genotype C relative to genotype B on 3, 4, 5, 7, and 8 DPI (*P* ≤ 0.003). There was a marginal decrease in average diarrhea scores of at least 0.53 units between genotype C relative to genotype B on 6 and 12 DPI (*P* ≤ 0.10). There were no differences in average diarrhea scores at 0, 1, 2, 9, and 14 DPI (*P *≥ 0.13) between genotypes A, B, or C.

Average lethargy scores were most severe in genotypes A and B compared to genotype C (Figure 3B). There was a significant reduction of at least 0.67 units in average lethargy scores between genotype C relative to genotype A on 3, 4, 5, 7, and 8 DPI (*P* ≤ 0.04). There was a marginally significant decrease of 0.86 units in average lethargy score between genotype C relative to genotype A on 6 DPI (*P* = 0.07). There was a significant reduction in average lethargy scores between genotypes B and C of at least 0.71 units on 4, 5, and 8 DPI (*P* ≤ 0.04). Additionally, there was a marginally significant decrease in average lethargy scores of at least 0.57 units on 3 and 7 DPI (*P* ≤ 0.08). There were no differences between genotypes A and B across any time points (*P* ≥ 0.10). There was no significant difference in lethargy scores on 0, 1, 2, 9, 10, 11, 12, 13, and 14 DPI (*P *≥ 0.11) between genotypes A, B, or C.

Average emaciation scores were most severe in genotypes A and B compared to genotype C (Figure 3C). There was a significant reduction in average emaciation score between genotype C relative to genotype A of at least 0.51 units on 3, 4, 5, 6, 7, 8, 11, and 12 DPI (*P* ≤ 0.05). Average emaciation scores were significantly reduced in genotype C relative to genotype B by at least 0.51 units on 3, 4, 5, 6, 7, 8, 11, and 12 DPI (*P* ≤ 0.05). Pigs in genotype C had a marginally reduced average emaciation score of 0.52 units compared to genotype B on 10 DPI (*P *= 0.09). There were no significant differences in average emaciation score between genotypes A, B, or C on 0, 1, 2, 9, 13, or 14 DPI (*P* ≥ 0.14).

### Growth Performance

Weights at placement (Table 1) were balanced across genotype ranging from 5.50 to 5.97 kg (*P* = 0.29). No differences were measured in end weights between genotypes on 3, 7, 14, 17, or 31 DPI among the surviving pigs (*≤*1.19 kg, *P* ≥ 0.34). Similarly, ADFI was not different among genotypes A, B, or C during any of the periods measured (*≤*164 g, *P *≥ 0.28).

**Table 1.** Effects of genotype on weanling pig growth performance after oral F18+ *Escherichia coli* inoculation

Item	Genotypes				
	A^1^	B^2^	C^3^	SEM	*P*-value^4^
Pig weights^5^, kg					
−3 DPI	5.5	6.0	6.0	0.2	0.29
3 DPI	6.3	6.3	6.5	0.3	0.95
7 DPI	6.6	6.6	7.3	0.4	0.37
14 DPI	9.4	9.5	9.6	0.7	0.98
17 DPI	10.6	10.6	11.3	0.8	0.80
31 DPI	17.5	18.1	18.7	1.0	0.73
ADG^6^, g/d					
−3 to 3 DPI	137^a^	149^a^	350^b^	39	<0.01
3 to 7 DPI	−46^a^	11^a^	503^b^	73	<0.01
7 to 14 DPI	671	726	825	99	0.56
3 to 17 DPI	451	597	766	86	0.07
17 to 31 DPI	1,070	1,172	1,193	71	0.46
−3 to 31 DPI	619^a^	657^ab^	841^b^	56	0.03
ADFI^6^, g/d					
−3 to 3 DPI	384	423	448	28	0.30
3 to 17 DPI	799	837	907	93	0.71
17 to 31 DPI	1,598	1,729	1,762	138	0.68
−3 to 31 DPI	1,011	1,027	1,139	85	0.53
G:F^6^					
−3 to 3 DPI	0.30^a^	0.39^a^	0.80^b^	0.10	0.01
3 to 17 DPI	0.55^a^	0.70^ab^	0.86^b^	0.07	0.02
17 to 31 DPI	0.68	0.68	0.70	0.06	0.97
−3 to 31 DPI	0.60^a^	0.66^ab^	0.74^b^	0.02	0.01

ADG = average daily gain; ADFI = average daily feed intake; DPI = days postinoculation; G:F = gain-to-feed ratio.

^1^A = genotype M307^GA^—heterozygous for F18+ *E. coli* susceptibility (*n* = 60 pigs, 5 pens, 12 pigs/pen).

^2^B = genotype M307^GG^—homozygous for F18+ *E. coli* susceptibility (*n* = 60 pigs, 5 pens, 12 pigs/pen).

^3^C = genotype M307^AA^—homozygous for F18+ *E. coli* resistance (*n* = 59 pigs, 5 pens, 11 to 12 pigs/pen).

^4^Treatments within a row that do not share a superscript differ (*P* < 0.05).

^5^Weights do not incorporate pig mortality between periods and represent surviving pigs only at each time point.

^6^ADG, ADFI, and G:F were corrected for mortality by adding weights of all pigs in the period and dividing by the cumulative days each pig was in the pen from the start of the weight period. A total of three pigs died from −3 to 3 DPI and 24 pigs from 3 to 7 DPI.

During the first dietary nursery phase (−3 to 3 DPI) and during the second nursery phase prior to intramuscular administration of enrofloxacin (3 to 7 DPI), ADG was significantly increased by at least 200 g in genotype C compared to both genotypes A and B (*P* < 0.01). There was a marginally significant increase (314 g, *P = *0.06) in ADG in genotype C compared to genotype A during the second nursery phase (3 to 17 DPI). Cumulatively (−3 to 31 DPI), the decreased performance during the first week translated into a 223 g increase in ADG in genotype C compared to genotype A (*P *= 0.04) and a marginally significant increase of 185 g compared to genotype B (*P *= 0.09). No differences in ADG were measured among genotypes A, B, or C during the third dietary phase (17 to 31 DPI, *P *≥ 0.47). Additionally, ADG was not different between genotype A and genotype B during any of the evaluated periods (*P *≥ 0.47).

Gain-to-feed ratios were significantly improved by at least 27% in genotype C compared to genotype A during the first (−3 to 3 DPI) and second (3 to 17 DPI) dietary phases (*P *≤ 0.02). This translated into significant 23% cumulative improvement in G:F between genotype C relative to genotype A (*P *< 0.01). No difference was measured in G:F between genotypes A and C during the third dietary phase (*P* = 1.00). There was a 105% improvement in G:F in genotype C compared to genotype B during the first nursery dietary phase (−3 to 3 DPI, *P *= 0.03) and a marginal improvement of 12% cumulatively (*P *= 0.07). Gain-to-feed ratios were not different between genotype A and genotype B during any of the dietary phases evaluated (*P *≥ 0.27).

### Molecular Characterization of Coliform Composition and Adherence in Ileal Scrapings

**Figure 4.**
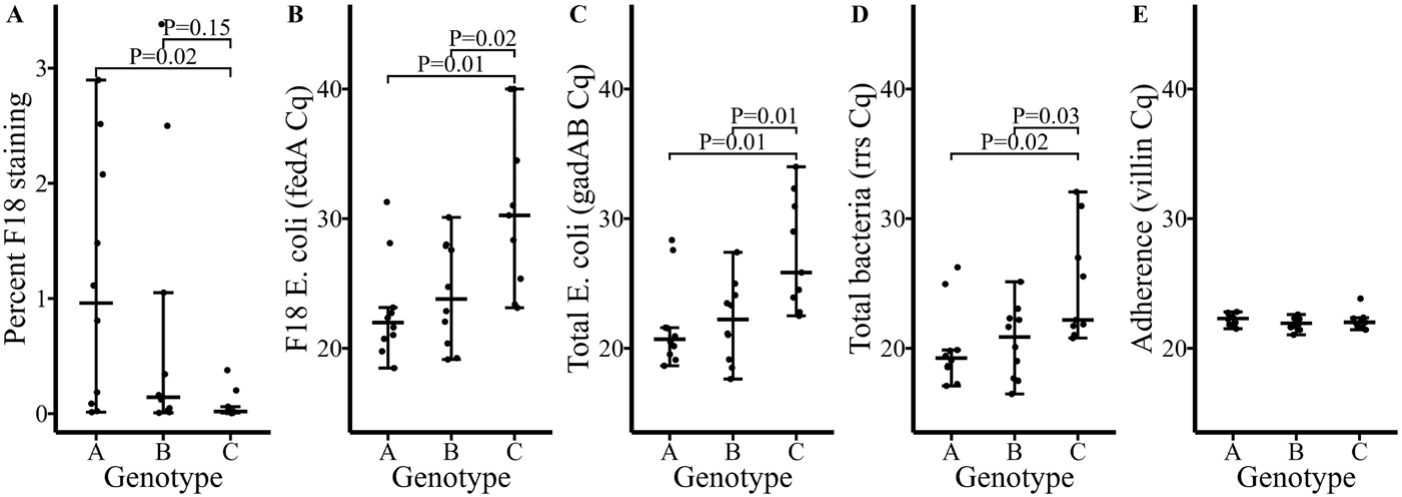
Molecular characterization of microbial nucleic acid loads collected from ileum scrapings at 3 DPI by (A) F18 ETEC-specific ISH or by rPCR for either (B) total F18 ETEC (fedA), (C) total *E. coli* (gadAB), (D) total bacteria (rrs), or (E) total adherent bacteria (villin) as measured by rPCR cycle threshold (Cq). Pigs sacrificed at 3 DPI from each genotype include A = genotype M307^GA^—heterozygous for *E. coli* susceptibility (*n* = 10, 5 pens, 2 pigs/pen), B = genotype M307^GG^—homozygous *E. coli* susceptibility (*n* = 10, 5 pens, 2 pigs/pen), and C = genotype M307^AA^—homozygous for *E. coli* resistance (*n* = 10, 5 pens, 2 pigs/pen). Pigs were orally inoculated with approximately 1 × 10^10^ CFU/mL for three consecutive days, starting at 0 DPI.

Ileal scrapings were evaluated for the presence of F18+ *E. coli* nucleic acid by both ISH (Figure 4A) and rPCR (fedA gene, Figure 4B). F18+ *E. coli*-specific nucleic acid staining was increased by 13.97-fold in genotype A (0.39%) compared to genotype C (0.03%, *P* = 0.02). These results corresponded to those found by F18-specific rPCR where quantitation cycle (Cq) values were increased by 7.74 in genotype C (Cq 30.66) compared to genotype A (22.92, *P <* 0.01). A 6.47 difference in F18+ *E. coli* Cq was found between genotype B (Cq: 24.20) compared to genotype C (30.66, *P* = 0.02) but there was no evidence of a difference by ISH (*P *= 0.15). No difference was measured between either genotype A or genotype B for F18+ *E. coli* between ISH or rPCR (*P *≥ 0.52).

In addition, rPCR was used to evaluate the presence of nucleic acid for total *E. coli* (gadAB gene, Figure 4C) total bacteria (rrs gene, Figure 4D), and total adherent bacteria (villin gene, Figure 4E). Genotype C had a 5.52 increase in total *E. coli* Cq compared to genotype A (*P *< 0.01) and similarly had a 4.71 increase in total bacteria Cq (*P *= 0.02). Genotype C also had a 5.24 increase in total *E. coli* Cq compared to genotype B (*P *= 0.01) and a 4.28 increase in total bacteria Cq (*P* = 0.03). No difference in either total *E. coli* or total bacteria Cqs was measured between genotype A and genotype B (*P* ≥ 0.94). No difference in adherent bacteria Cq was measured between genotypes A, B, or C (*P *≥ 0.38).

## DISCUSSION

The results of this study demonstrated that pigs with the homozygous resistant genotype had greater survivability, reduced clinical signs, and improved growth performance relative to both susceptible genotypes. The challenge isolate used in this study caused severe clinical disease in both susceptible genotypes through the expression of a heat-labile toxin, heat-stabile toxin B, Shiga-like toxin II, and heat-stabile enterotoxin. The performance and clinical data were supported by ISH and rPCR in ileal scrapings at 3 DPI, which demonstrated decreased F18 attachment leading to decreased total *E. coli* and total bacteria in the mucosa.

This work is supported by previous studies evaluating genetic resistance against *E. coli* infection. Fimbrial-receptor interaction is crucial for the pathogenesis of postweaning colibacillosis. Resistance against F18+ *E. coli* infection has been associated with genetic polymorphisms that are thought to interfere with bacterial attachment to host receptors. Initial studies suggested adherence by the K88 (F4) fimbrial type was moderated by two host alleles with adherence dominating over nonadherence (Sellwood et al., 1974; Rutter et al., 1975). The α(1,2) fucosyltransferases (*FUT1* and *FUT2*) mapped to porcine chromosome 6 are hypothesized to be important for controlling F18+ *E. coli* fimbrial adhesion (Meijerink et al., 1997). There have been several *FUT1* polymorphisms proposed to have a possible influence on F18+ *E. coli* adhesion including at position 103 (Ala → Thr) and position 286 (Arg → Glu) (Meijerink et al., 2000).

An F18 challenge study conducted by Frydendahl et al. (2003) evaluated the base pair 307 mutation (M307, position 286) with genotype M307^AA^ representing resistance and the M307^GG^ and M307^AG^ representing susceptibility to F18+ *E. coli* colonization. They demonstrated a statistical association with host resistance to F18+ *E. coli* infection with genotype. Fewer resistant pigs developed diarrhea compared to the susceptible pigs. However, a minority of resistant pigs developed mucosal colonization, indicating incomplete protection associated with the receptor genotype. Additional genes may contribute to host resistance against F18+ *E. coli* infection. Work by Dong et al. (2016) found a significant association between *FUT2* and F18+ *E. coli* resistance. However, they also found other glycosphingolipid biosynthesis-globo series pathway genes such as *FUT1*, ST3GAL1, and B3GALNT1 may also participate in resistance.

Enrofloxacin (Baytril, Elanco, Greenfield, IN) was administered intramuscularly to all animals at 7 DPI at a dose of 7.5 mg/kg due to the virulent nature of the challenge isolate and a concern for the welfare of the inoculated pigs. The treatment was effective at reducing mortality as only one mortality occurred in the resistant groups after 7 DPI. Fluoroquinolones and third-generation cephalosporins are important drug class in human medicine and are therefore heavily regulated (Fairbrother and Nadeau, 2019). Pigs may represent a reservoir for resistant *E.* coli, which potentially could transfer AMR genes to bacteria of human importance by means of horizontal gene transfer (O’Neill et al., 2023). The effectiveness of antimicrobials to control *E. coli* has reduced in recent years as AMR has increased. From 2013 to 2019, data from the National Antimicrobial Resistance Monitoring System (NARMS) demonstrated an increasing linear trend in F18+ *E. coli*-associated AMR for many antibiotics including amoxicillin-clavulanic acid, ampicillin, azithromycin, cefoxitin, ceftriaxone, and trimethoprim-sulfamethoxazole (Sodagari and Varga, 2023). In addition, data from the Iowa State University Veterinary Diagnostic Laboratory also show a problematic trend as AMR toward enrofloxacin has increased in F18+ *E. coli* isolates obtained from affected pigs since 2019 (Paiva et al., 2024).

Knowledge of the animals’ genotype for F18+ *E. coli* resistance can direct mating strategies as well as treatment and feeding strategies. Breeding of genetically resistant pigs to F18+ *E. coli* infection may be one way to reduce antimicrobial use and decrease AMR against drug classes of human importance. The results of this study showed resistance against one F18+ *E. coli* isolates. However, creating breeding schemes for F18-resistant pigs may have substantial challenges. F18-positive isolates have greater genetic diversity than F4-positive isolates and have been assigned to at least six clonal groups (García et al., 2020). It is unclear whether there will be cross-resistance to genetically divergent F18 isolates, and therefore this work needs to be replicated with other strains. In addition, resistance to F18+ *E. coli* infection has been associated with multiple genetic loci including *FUT1*, *FUT2*, ST3GAL1, and B3GALNT (Meijerink et al., 1997; Dong et al., 2016). It will also be important to avoid co-selection of unwanted traits closely linked with loci coding for the F4 and F18 receptors. It is also unknown whether additional fimbrial types or new variants will emerge that could bind to yet unidentified receptors (Fairbrother and Nadeau, 2019). Despite these challenges, this study provides strong support for the use of genetic selection for F18+ *E. coli* resistance for controlling PWD and warrants further investigation.

## References

[CIT0001] Amezcua, R., R. M. Friendship, C. E. Dewey, C. Gyles, and J. M. Fairbrother. 2002. Presentation of postweaning *Escherichia coli* diarrhea in southern Ontario, prevalence of hemolytic *E. coli* serogroups involved, and their antimicrobial resistance patterns. Can. J. Vet. Res. 66:73–78. 11989737 PMC226986

[CIT0002] Arruda, B., P. Piñeyro, R. Derscheid, B. Hause, E. Byers, K. Dion, D. Long, C. Sievers, J. Tangen, T. Williams, et al 2019. PCV3-associated disease in the United States swine herd. Emerg. Microbes Infect. 8:684–698. doi: https://doi.org/10.1080/22221751.2019.1613176 31096848 PMC6534263

[CIT0003] Becker, S. L., Q. Li, E. R. Burrough, D. Kenne, O. Sahin, S. A. Gould, and J. F. Patience. 2020. Effects of an F18 enterotoxigenic *Escherichia coli* challenge on growth performance, immunological status, and gastrointestinal structure of weaned pigs and the potential protective effect of direct-fed microbial blends. J. Anim. Sci. 98:1–10. doi: https://doi.org/10.1093/jas/skaa113 PMC7228676 32300795

[CIT0004] Coddens, A., F. Verdonck, P. Tiels, K. Rasschaert, B. M. Goddeeris, and E. Cox. 2007. The age-dependent expression of the F18+ *E. coli* receptor on porcine gut epithelial cells is positively correlated with the presence of histo-blood group antigens. Vet. Microbiol. 122:332–341. doi: https://doi.org/10.1016/j.vetmic.2007.02.007 17353102

[CIT0005] Deprez, P., C. V. D. Hende, E. Muylle, and W. Oyaert. 1986. The influence of the administration of sow’s milk on the postweaning excretion of hemolytic *E. coli* in the pig. Vet. Res. Commun. 10:469–478. doi: https://doi.org/10.1007/BF02214010 3541366

[CIT0006] Dong, W. H., C. H. Dai, L. Sun, J. Wang, S. Y. Sun, G. Q. Zhu, S. L. Wu, and W. B. Bao. 2016. Expression of key glycosphingolipid biosynthesis-globo series pathway genes in *Escherichia coli* F18-resistant and *Escherichia coli* F18-sensitive piglets. Anim. Genet. 47:428–435. doi: https://doi.org/10.1111/age.12428 26970430

[CIT0007] Duarte, M. E., J. Tyus, and S. W. Kim. 2020. Synbiotic effects of enzyme and probiotics on intestinal health and growth of newly weaned pigs challenged with enterotoxigenic F18(+) *Escherichia coli*. Front. Vet. Sci. 7:573. doi: https://doi.org/10.3389/fvets.2020.00573 33033721 PMC7509054

[CIT0008] Euken, R., and L. Schulz. 2021. Assessing economic opportunity of improving mortality rate in wean-to-finish swine production. [accessed July 9, 2024]. https://www.extension.iastate.edu/agdm/livestock/html/b1-78.html.

[CIT0009] Fairbrother, J. M., and E. Nadeau. 2019. Colibacillosis. In: Zimmerman, J. J., L. A. Karriker, A. Ramirez, K. J. Schwartz, G. W. Stevenson, and J. Zhang, editors. Diseases of swine. Hoboken (NJ): John Wiley & Sons, Inc.; p. 807–834.

[CIT0010] Fairbrother, J. M., E. Nadeau, and C. L. Gyles. 2005. *Escherichia coli* in postweaning diarrhea in pigs: an update on bacterial types, pathogenesis, and prevention strategies. Anim. Health Res. Rev. 6:17–39. doi: https://doi.org/10.1079/ahr2005105 16164007

[CIT0011] Frydendahl, K., T. Kare Jensen, J. Strodl Andersen, M. Fredholm, and G. Evans. 2003. Association between the porcine *Escherichia coli* F18 receptor genotype and phenotype and susceptibility to colonisation and postweaning diarrhoea caused by *E. coli* O138:F18. Vet. Microbiol. 93:39–51. doi: https://doi.org/10.1016/s0378-1135(02)00348-6 12591205

[CIT0012] García, V., M. Gambino, K. Pedersen, S. Haugegaard, J. E. Olsen, and A. Herrero-Fresno. 2020. F4- and F18-positive enterotoxigenic *Escherichia coli* isolates from diarrhea of postweaning pigs: genomic characterization. Appl. Environ. Microbiol. 86:e01913-20. doi: https://doi.org/10.1128/AEM.01913-20 PMC7657637 32948526

[CIT0013] Hansen, S. V., N. Canibe, T. S. Nielsen, and T. A. Woyengo. 2024. Zinc status and indicators of intestinal health in enterotoxigenic *Escherichia coli* F18 challenged newly weaned pigs fed diets with different levels of zinc. J. Anim. Sci. 102:skae018. doi: https://doi.org/10.1093/jas/skae018 38245836 PMC10939430

[CIT0014] Hur, J., and J. H. Lee. 2013. Protection against neonatal *Escherichia coli* diarrhea by vaccination of sows with a novel multivalent vaccine candidate expressing *E. coli* adhesins associated with neonatal pig colibacillosis. Res. Vet. Sci. 94:198–204. doi: https://doi.org/10.1016/j.rvsc.2012.08.004 22959394

[CIT0015] Jang, K. B., V. H. C. Moita, N. Martinez, A. Sokale, and S. W. Kim. 2023. Efficacy of zinc glycinate reducing zinc oxide on intestinal health and growth of nursery pigs challenged with F18+ *Escherichia coli*. J. Anim. Sci. 101:skad035. doi: https://doi.org/10.1093/jas/skad035 36715157 PMC10195191

[CIT0016] Lessard, M., M. Dupuis, N. Gagnon, E. Nadeau, J. J. Matte, J. Goulet, and J. M. Fairbrother. 2009. Administration of *Pediococcus acidilactici* or *Saccharomyces cerevisiae boulardii* modulates development of porcine mucosal immunity and reduces intestinal bacterial translocation after *Escherichia coli* challenge. J. Anim. Sci. 87:922–934. doi: https://doi.org/10.2527/jas.2008-0919 19028865

[CIT0017] Meijerink, E., R. Fries, P. Vögeli, J. Masabanda, G. Wigger, C. Stricker, S. Neuenschwander, H. U. Bertschinger, and G. Stranzinger. 1997. Two alpha(1,2) fucosyltransferase genes on porcine chromosome 6q11 are closely linked to the blood group inhibitor (S) and *Escherichia coli* F18 receptor (ECF18R) loci. Mamm. Genome. 8:736–741. doi: https://doi.org/10.1007/s003359900556 9321466

[CIT0018] Meijerink, E., S. Neuenschwander, R. Fries, A. Dinter, H. U. Bertschinger, G. Stranzinger, and P. Vögeli. 2000. A DNA polymorphism influencing alpha(1,2)fucosyltransferase activity of the pig FUT1 enzyme determines susceptibility of small intestinal epithelium to *Escherichia coli* F18 adhesion. Immunogenetics 52:129–136. doi: https://doi.org/10.1007/s002510000263 11132149

[CIT0019] O’Neill, L., E. G. Manzanilla, D. Ekhlas, and F. C. Leonard. 2023. Antimicrobial resistance in commensal *Escherichia coli* of the porcine gastrointestinal tract. Antibiotics (Basel). 12:1616. doi: https://doi.org/10.3390/antibiotics12111616 37998818 PMC10669415

[CIT0020] Paiva, R. C., E. R. Burrough, and M. Almeida. 2024. Characterization of hemolytic *E. coli* and antimicrobial susceptibility from ISU-VDL submitted cases from 2010 to 2022. In: AASV Annual Meeting 2024, Nashville, TN.

[CIT0021] Ruan, X., M. Liu, T. A. Casey, and W. Zhang. 2011. A tripartite fusion, FaeG-FedF-LT(192)A2:B, of enterotoxigenic *Escherichia coli* (ETEC) elicits antibodies that neutralize cholera toxin, inhibit adherence of K88 (F4) and F18 fimbriae, and protect pigs against K88ac/heat-labile toxin infection. Clin. Vaccine Immunol. 18:1593–1599. doi: https://doi.org/10.1128/CVI.05120-11 21813665 PMC3187021

[CIT0022] Rutter, J. M., M. R. Burrows, R. Sellwood, and R. A. Gibbons. 1975. A genetic basis for resistance to enteric disease caused by *E. coli*. Nature 257:135–136. doi: https://doi.org/10.1038/257135a0 1099454

[CIT0023] Sellwood, R., R. A. Gibbons, G. W. Jones, and J. M. Rutter. 1974. A possible basis for the breeding of pigs relatively resistant to neonatal diarrhoea. Vet. Rec. 95:574–575. doi: https://doi.org/10.1136/vr.95.25-26.574 4615428

[CIT0024] Smith, B. N., M. Hannas, C. Orso, S. Martins, M. Wang, S. M. Donovan, and R. N. Dilger. 2020. Dietary osteopontin-enriched algal protein as nutritional support in weaned pigs infected with F18-fimbriated enterotoxigenic *Escherichia coli*. J. Anim. Sci. 98:1–15. doi: https://doi.org/10.1093/jas/skaa314 PMC7584275 32954424

[CIT0025] Sodagari, H. R., and C. Varga. 2023. Evaluating antimicrobial resistance trends in commensal *Escherichia coli* isolated from cecal samples of swine at slaughter in the United States, 2013-2019. Microorganisms 11:1033. doi: https://doi.org/10.3390/microorganisms11041033 37110456 PMC10142105

[CIT0026] Sun, Y., M. E. Duarte, and S. W. Kim. 2021. Dietary inclusion of multispecies probiotics to reduce the severity of post-weaning diarrhea caused by *Escherichia coli* F18(+) in pigs. Anim. Nutr. 7:326–333. doi: https://doi.org/10.1016/j.aninu.2020.08.012 34258420 PMC8245796

[CIT0027] Van den Broeck, W., E. Cox, and B. M. Goddeeris. 1999. Receptor-dependent immune responses in pigs after oral immunization with F4 fimbriae. Infect. Immun. 67:520–526. doi: https://doi.org/10.1128/IAI.67.2.520-526.1999 9916054 PMC96350

[CIT0028] White, C. S., C. C. Hung, S. Lanka, C. W. Maddox, A. Barri, A. O. Sokale, and R. N. Dilger. 2024. Dietary monoglyceride supplementation to support intestinal integrity and host defenses in health-challenged weanling pigs. J. Anim. Sci. 102:1–13. doi: https://doi.org/10.1093/jas/skae105 PMC11044705 38629856

